# Two promising natural lipopeptides from *Bacillus subtilis* effectively induced membrane permeabilization in *Candida glabrata*

**DOI:** 10.3389/abp.2024.11999

**Published:** 2024-01-31

**Authors:** Madhuri Madduri, Shivaprakash M. Rudramurthy, Utpal Roy

**Affiliations:** ^1^ Department of Biological Sciences, BITS Pilani K.K. Birla Goa Campus, Vasco da Gama, Goa, India; ^2^ Department of Medical Microbiology, Medical Mycology Division, Post Graduate Institute of Medical Education and Research (PGIMER), Chandigarh, India

**Keywords:** antifungal, bioactive, *Bacillus subtilis*, *Candida glabrata*, lipopeptides, membrane permeabilization

## Abstract

*Candida glabrata* is an important opportunistic human pathogen well known to develop resistance to antifungal drugs. Due to their numerous desirable qualities, antimicrobial lipopeptides have gained significant attention as promising candidates for antifungal drugs. In the present study, two bioactive lipopeptides (AF_4_ and AF_5_ m/z 1071.5 and 1085.5, respectively), coproduced and purified from *Bacillus subtilis* RLID12.1, consist of seven amino acid residues with lipid moieties*.* In our previous studies, the reversed phased-HPLC purified lipopeptides demonstrated broad-spectrum of antifungal activities against over 110 *Candida albicans, Candida* non*-albicans* and mycelial fungi. Two lipopeptides triggered membrane permeabilization of *C. glabrata* cells, as confirmed by propidium iodide-based flow cytometry, with PI uptake up to 99% demonstrating fungicidal effects. Metabolic inactivation in treated cells was confirmed by FUN-1-based confocal microscopy. Together, the results indicate that these lipopeptides have potentials to be developed into a new set of antifungals for combating fungal infections.

## Introduction

After *Candida albicans*, the most prevalent yeast species reported to have been isolated from humans is *Candida glabrata* (*C. glabrata*). The past two and a half decades have witnessed *C*. *glabrata* becoming one of the most predominant *Candida* species, causing fungal infections (Chakrabarti et al., 2009; Lockhart et al., 2012; Benaducci et al., 2014; Cleveland et al., 2015; Pappas et al., 2018; Naicker et., 2023). The reason behind this steep upward trend of *C. glabrata* infections may be associated with geographical variation and the rampant overuse of azoles (Richardson and Lass-Flörl, 2008). Given the prevalence of *C. glabrata* as the second leading cause of candidiasis infections, including oral candidiasis, candidemia, invasive candidiasis, and urinary candidiasis (Esfandiary et al., 2012; Nash et al., 2016), it is essential to understand the alarming 40% mortality rate associated with *C. glabrata* infection underscores the seriousness of this opportunistic human pathogen, especially in immunocompromised hosts (Rodrigues et al., 2014; Mota et al., 2015; Nagayoshi et al., 2017). It has been recently reported that *C. glabrata* has developed several strategies to survive and thrive in its host cells and found ways to overcome antifungal resistance to commonly used antifungals, which also contribute to increased virulence (Pappas et al., 2018; Salazar et al., 2018). Flow cytometry (FCM) has been one of the major techniques that has successfully demonstrated its utility in determining the effect of antimycotics on yeast cells, cell membranes, or mechanisms of action when used with appropriate dye or fluorescent probes and experimental conditions (Pore, 1994). In our previous study (Ramachandran et al., 2018b), the antifungal susceptibility test (AFST) analyses of the two lipopeptides were done according to the CLSI guidelines. In this current investigation, the two lipopeptide fractions (AF_4_ and AF_5_) coproduced by *Bacillus subtilis* RLID 12.1 were purified, and the main objective of the study was to determine their membrane-permeabilizing effects on the yeast cell membrane and metabolic activity at two different peptide concentrations using the membrane-impermeant propidium iodide (PI) dye and two-colour FUN-1 stain.

## Materials and methods

### Microorganisms

The test strain *C. glabrata* ATCC 2001 was obtained from the National Culture Collection of Pathogenic Fungi (NCCPF), Post Graduate Institute of Medical Education and Research (PGIMER) Chandigarh, India and maintained as 20% glycerol stock at −80°C. The producer strain was also maintained as glycerol stock at −80°C.

### Extraction and purification of antifungal compounds

For the purification of antifungal lipopeptides, cell-free supernatant (CFS) was obtained after 60 h at 30°C of incubation under stirring conditions of cells of the strain *B. subtilis* RLID 12.1. The CFS was subjected to a three-step purification that included HCl precipitation, solvent extraction using n-butanol, and silica gel (mesh size 230–400) based adsorption chromatography using proper ratios of methanol and chloroform. After the adsorption chromatography, all the collected fractions were tested for antifungal activity by spot-on-lawn assay against freshly grown *C. glabrata* cells spreading on Sabourad dextrose agar (SDA) plates. The 5 μL aliquots of fractions which showed the clear zones of inhibition after 24 h of incubation were considered as bioactive fractions. These bioactive fractions AF_4_ and AF_5_ were fractionated using the reversed phased-HPLC system (Agilent Technologies, United States) at a semi-preparative scale equipped with a variable wavelength detector and an Agilent C18 column (10 mm × 250 mm, 5 μm) (Ramachandran et al., 2018a; Ramachandran et al., 2018b). The analytical scale HPLC profiles of AF_4_ and AF_5_ and their m/z values are shown in Supplementary Figure S1.

### Antifungal susceptibility testing (AFST) against *Candida glabrata*

The minimum inhibitory concentrations (MICs) of the purified antifungal lipopeptides AF_4_ and AF_5_ were tested against *C. glabrata* ATCC 2001, according to CLSI guidelines (M27-A2) (Clinical and Laboratory Standards Institute, 2008). For antifungal assays, two-fold serial dilutions of each drug were prepared in RPMI-1640 (pH 7.0 ± 0.1) (with L-glutamine and phenol red without sodium bicarbonate) was buffered with 0.165 M MOPS (morpholinepropanesulfonic acid) (Himedia) and supplemented with 0.2% glucose. As reference yeast strain for the AFST, *C. albicans* ATCC 24433 and the positive control amphotericin B (AMB) (HiMedia, India) were used.

### PI uptake assay by flow cytometry (FCM)

To study the impact of novel antifungal lipopeptides on membrane integrity, lipopeptides AF_4_ and AF_5_ (8 and 16 mg/L), and AMB (×1 and ×2 MIC) were added to *Candida* cell suspension (1 × 10^6^ CFU/mL) in RPMI 1640 and incubated at 37°C for 18 and 5 h, respectively, in shaking conditions (Gokahmetoglu et al., 2003; Seyedjavadi et al., 2020). Two types of controls were used in the study: negative controls consisting of untreated cells, and PI positive controls, treated with 70% ethanol for 30 min. Post-incubation, the cells were harvested, washed with 1× phosphate buffered saline (PBS), and stained with PI at a concentration of 7.5 μg/mL for 20 min. Unstained and untreated cells were sampled and analysed in the beginning. Flow cytometry analysis was performed on 30,000 events using a FACSMelody flow cytometer with a 488 nm laser line and a 586 nm filter with PI detection.

### Viability (CFU) assays

Prior PI staining, aliquots of yeast cultures (both untreated and treated) were collected, serially diluted in sterile 1×PBS, and plated in duplicate on SDA plates (Chaturvedi et al., 2004; Ramesh et al., 2023). The plates were incubated for 24 h at 37°C, colonies were counted, and findings were represented as the percentage reduction in CFU/mL compared to the growth in the untreated sample.

### Confocal laser scanning microscopy (CLSM)

The CLSM analysis was performed using PI (ThermoFisher, United States) and [2-chloro-4-(2, 3-dihydro-3-methyl-(benzol-1, 3-thiazol-2-yl)-methylidene)-1-phenylquinolininum iodide] FUN-1 (ThermoFisher, United States) (Chan et al., 2011; Zhang et al., 2018). Cells were treated with AF_4_/AF_5_ (8 and 16 mg/L) for 18 h, AMB (1 mg/L) for 5 h, and untreated cells were used as control. Post-treatments, cells were harvested and resuspended in 1×PBS, following which staining with PI (7.0 μg/mL) for 20 min in the dark and washing with 1×PBS were done. FUN-1 at a final concentration of 5 μM was prepared in 10 mM glucose-HEPES (GH) buffer, and cells were incubated in (GH) buffer with FUN-1 stain for 30 min (Pina-Vaz et al., 2001; Kwolek-Mirek and Zadrag-Tecza, 2014; Yan et al., 2019). Treated and untreated cells were imaged with an Olympus FV3000 (Japan) microscope at 60 × 2 and 100 × 2 magnifications for PI-stained cells and 100 × 2 for FUN-1 stained cells.

### Statistical analysis

Experiments were performed in three individual times with two technical replicates. Data are presented as the means ±SD values and, One-way ANOVA statistical analysis was done in the Graph-pad prism Software version 9.3.1.

## Results and discussion

**FIGURE 1:**
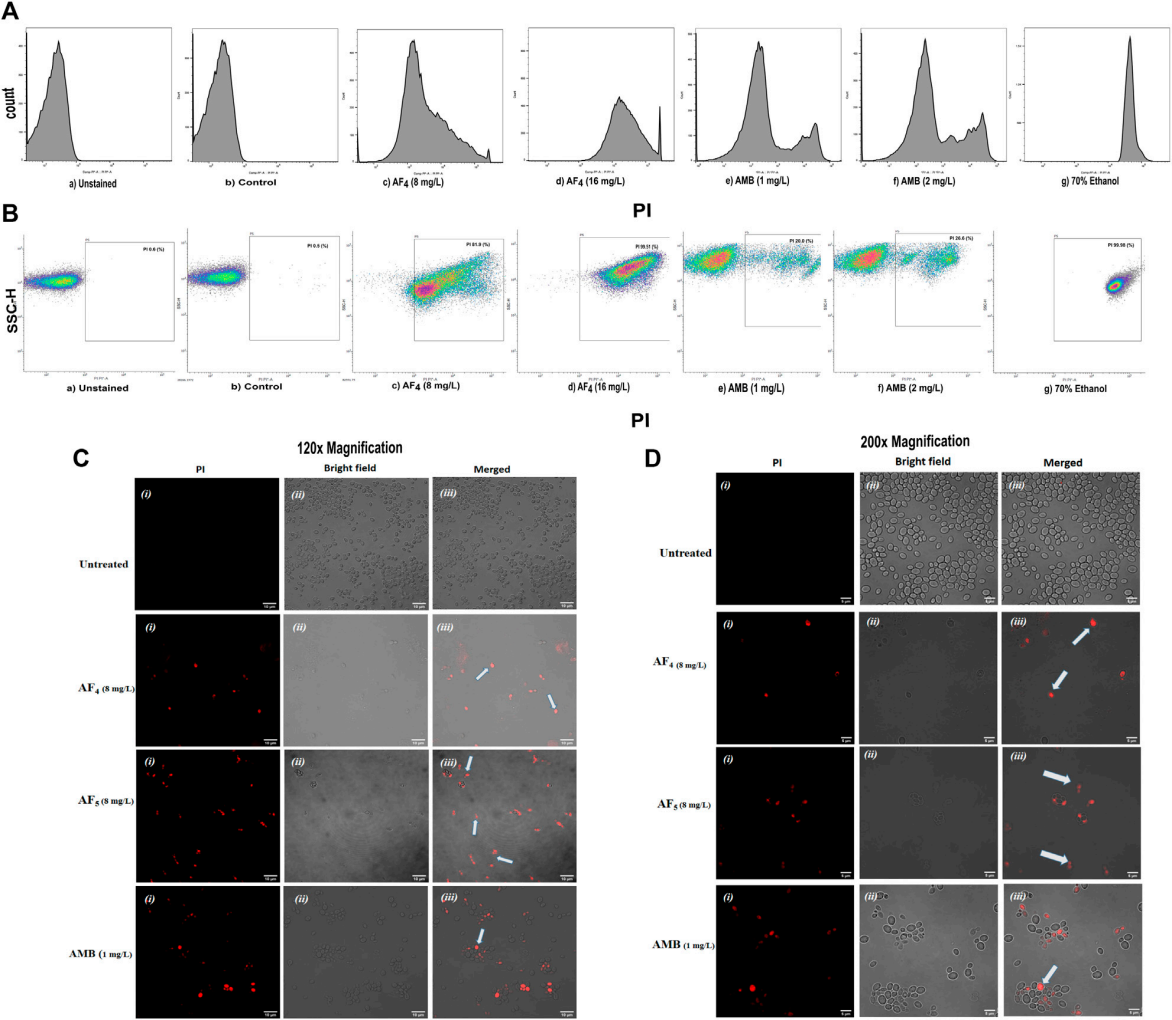
The effect of the lipopeptide AF_4_ on the cell membrane permeability of *C. glabrata* 2001, measured by PI uptake. Experiments recorded forward scatter, side scatter, and the percentage of PI-stained cells, and the data was analyzed using the software FlowJo version 10.8.1. The histograms related to PI fluorescence intensity were split into two quadrants, i.e., one with PI negative cells (viable cells), and another with PI-positive cells (membrane compromised cells). **(A)** Flow cytometry analysis of membrane permeabilization assay by PI uptake. **(B)** The side scatter versus PI plot shows the shift of population of PI-positive cells along the x-axis. **(C,D)** Confocal images of *C. glabrata* cell membrane integrity treated with AF_4_, AF_5_ and AMB monitored by PI uptake (Left panel, magnification 60 × 2 and, right panel 100 × 2, respectively). A few cells in the field as indicated by white arrows are non-viable.

**FIGURE 2:**
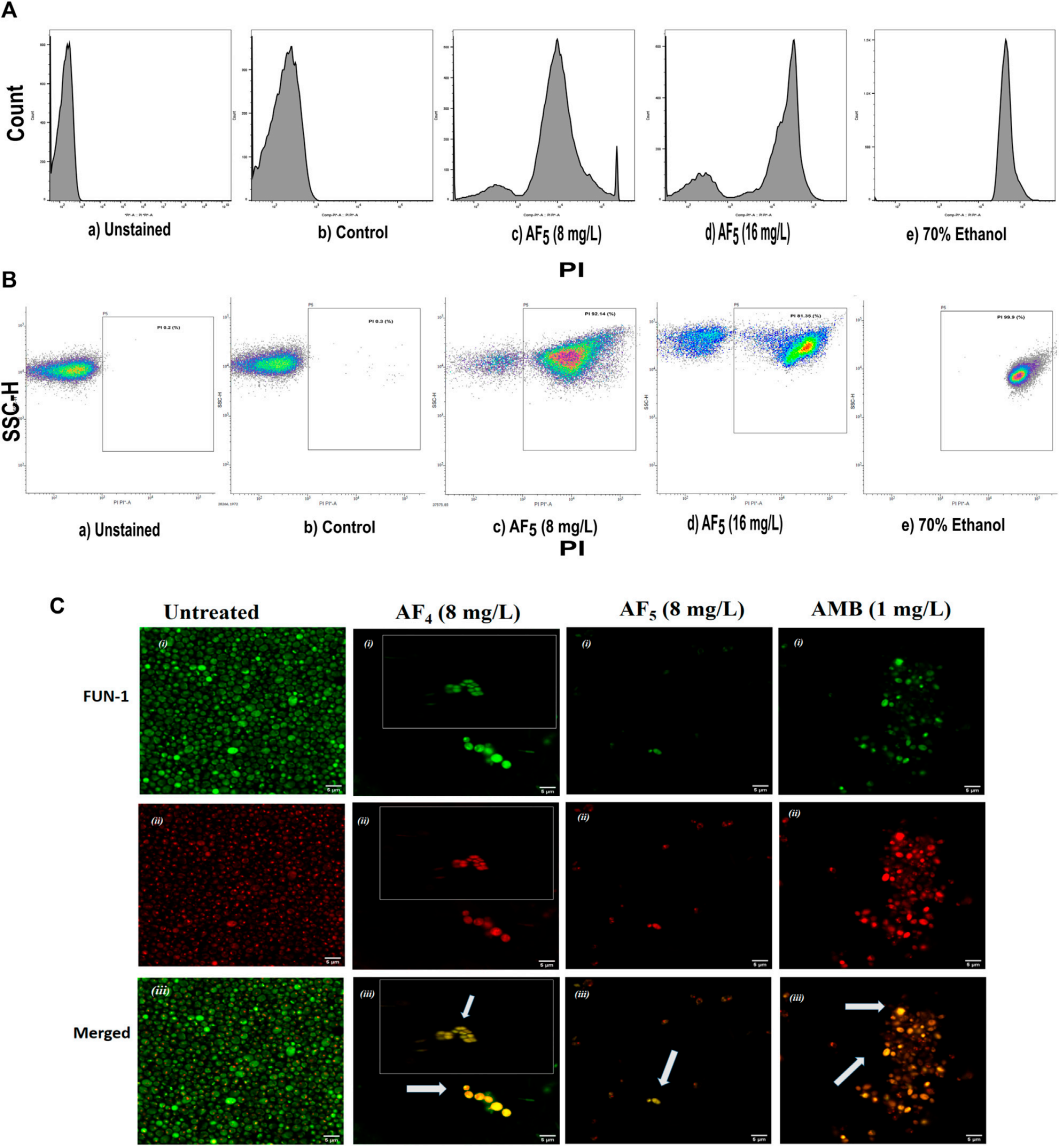
The effect of the lipopeptide AF_5_ on the cell membrane permeability of *C. glabrata* 2001, determined by PI uptake. **(A)** Flow cytometry analysis of membrane permeabilization assay. Cells were treated with AF_5_ (8) and (16) mg/L compared with the control (untreated). **(B)** The side scatter versus PI plot shows the shift of population of PI-positive cells along the x-axis. Density plot of PI uptake by *C. glabrata* cell populations of a) unstained, b) control, and c) and d) AF_5_ (8) and (16) mg/L treated cells, respectively, along with e) 70% ethanol. Ethanol-treated cells exhibited a high permeability to PI (99.99% cells stained). **(C)** Confocal image (×200 magnification) showing cells stained with FUN-1, the AF_4_, AF_5_ and AMB treated cells are without discernible and red fluorescent CIVS and show yellow-green fluorescence in the merged channel, representing metabolically inactive cells compared to untreated cells. The inset images show non-viable cells from another field.

The MICs of two lipopeptides and AMB (used as the standard antifungal) against *C. glabrata* were determined to be 4 mg/L and 1 mg/L, respectively. The results agree with the previously determined MIC values (Ramachandran et al., 2018b; Ramchandran et al., 2020). To gain more insights into lipopeptides’ membrane permeabilizing potential, we performed PI uptake assays using 8 mg/L (×2) and 16 mg/L (×4) MICs as determined by the AFST for *C*. *glabrata* ATCC 2001. FC results were expressed as the fluorescence intensity (FI) of PI-stained yeasts. AF_4_ and AF_5_ treatments caused cell membrane disruption in *C. glabrata* cells, as seen by the enhanced fluorescence that resulted from PI uptake. In cells treated with AMB, AF_4_, AF_5_, and 70% ethanol, the increase in PI-fluorescence is manifested as a distinct shift of the peak along the x-axis (Figures 1A, B) and (Figures 2A, B). The histograms pertaining to all flow cytometry assays where *C. glabrata* ATCC 2001 cells were stained with PI after being exposed to AMB for 5 h (Gokahmetoglu et al., 2003) at 1 and 2 mg/L and AF_4_/AF_5_ (8 and 16 mg/L) for 18 h have been shown in Figures 1A, 2A. All the events of FC population density plots have been shown in Figures 1B, 2B.

**FIGURE 3:**
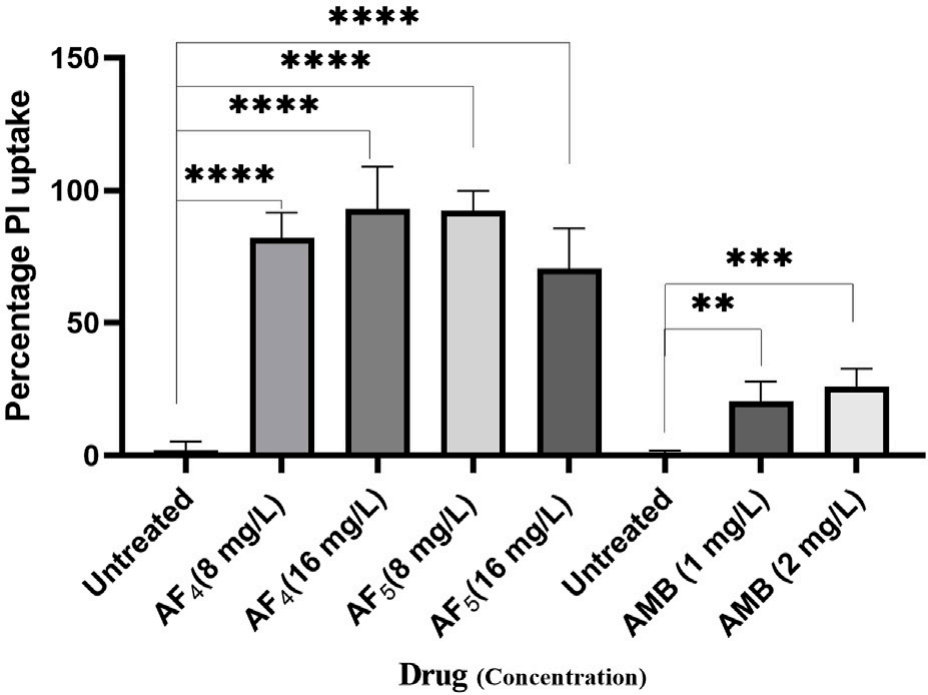
The histogram analysis in the graph shows the percentage of PI-positive *C. glabrata* cells upon (AF_4_/AF_5_) antifungal treatments. Ethanol-treated yeast cells exhibited a high permeability to PI (99.99% of cells stained). P-values (****p < 0.0001) indicate statistically significant differences from the effects of AF_4_ and AF_5_ -treatments and control (untreated). Statistically significant observations were made between AMB-treated cells and control used during AMB-treatments.

Significant congruence was found when comparing the proportion of PI uptake with the drop in CFU/mL among AF_4_ treatments. A substantial association was observed between the percentage PI uptake estimated in flow cytometry and the percentage reduction in plate counts, presented in tabular form (Table 1) to summarize the findings. Cells unexposed to lipopeptides showed very negligible fluorescence, as evidenced by the PI uptake percentage graph (Figure 3). At 8 mg/L, the AF_4_-treated *C. glabrata* showed an average of 81.92% PI-positive cells and 99.90% CFU reduction, better was the observation in AF_4_-(16 mg/L)-treated *C. glabrata* cells, where 99.51% PI-positive cells were noted and a CFU reduction of 99.97% recorded (Figure 3; Table 1). At 8 mg/L, the AF_5_-treated *C. glabrata* showed an average of 92.14% PI-positive cells and 99.4% CFU reduction. However, AF_5_-(16 mg/L)-treated *C. glabrata* cells showed 81.35% PI-positive cells, and the CFU reduction of 99.92% was recorded, indicating that AF_5_ even at a 2-fold concentration, did not show more membrane permeabilization and increased fungicidal effects. This may be due to the hydrophobic aggregation of AF_5_ lipopeptide at higher concentrations (Ramchandran et al., 2020). In AMB (1 and 2 mg/L)-treated cells for 5 h, only 20.02% and 26.06% PI-positive cells were detected, respectively. The increase in the fluorescence intensity percentage observed in yeast cells treated with higher AF_4_ and AF_5_ (8 mg/L) concentrations agreed with the positive control values, indicating potential antifungal activity. In comparison, untreated cells showed negligible fluorescence.

**TABLE 1:** Correlation between PI uptake percentage from flow cytometry and plate count.

Drug (concentration)	PI uptake (%)	Plate count	
		Log reduction	Reduction (%)
AF_4_ (8 mg/L)	81.92 ± 8.7 SD	3.03	99.90 ± 0.79 SD
AF_4_ (16 mg/L)	99.51 ± 0.07 SD	3.64	99.97 ± 0.59 SD
AF_5_ (8 mg/L)	92.14 ± 6.8 SD	2.25	99.44 ± 0.82 SD
AF_5_ (16 mg/L)	81.35 ± 12.8 SD	3.10	99.92 ± 2.1 SD
AMB (1 mg/L)	20.02 ± 6.7 SD	4.12	99.99 ± 0.53 SD
AMB (2 mg/L)	26.06 ± 6.02 SD	4.43	99.99 ± 0.56 SD

*Candida* cells exposed to AF_4_/AF_5_ (8 mg/L) displayed PI uptake as evident from red fluorescence, indicating cell membrane damage and hence permeabilization [Figure 1C (×120) and Figure 1D (×200)] magnifications. Since the formation of cylindrical intravacuolar structures (CIVS) needs metabolically active cells with undamaged plasma membrane, the presence of yellow-green fluorescence (Figure 2C) in FUN-1 stained cells instead of red fluorescence in lipopeptide-treated cells clearly indicates the loss of membrane integrity, metabolic activity, and cell death (Millard et al., 1997). The cell deaths are quite evident from the log reductions of 3.03 and 3.64 CFU/mL by AF_4_ (8 and 16 mg/L). In the lipopeptide-treated and AMB-treated cells CIVS were not observed. Collectively, it may be surmised that the prime targets of AF_4_ and AF_5_ might be the components of the cell membrane (Ramesh et al., 2023). The presence of a long acyl chain in antifungal cyclic lipopeptides such as iturin and fenzycin enables oligomer formation and subsequent insertion into the plasma membrane (Malina and Shai, 2005) and the organisation of aggregates in biological membranes triggering early membrane damage and membrane disintegration was also reported by Horn et al. (2013). The preliminary mechanism of action on the yeast cell membrane has been schematically depicted in the Supplementary Figure S2. Taken together, the results revealed that two lipopeptides demonstrated remarkable fungicidal effects on *C. glabrata* cells with enhanced cell membrane permeability, and damage. Consequently, the findings positively indicate that the lipopeptides AF_4_ and AF_5_ hold considerable promise as prospective antifungal agents.

## Data Availability

The raw data supporting the conclusion of this article will be made available by the authors, without undue reservation.

## References

[B1] Benaducci T. Matsumoto M. T. Sardi J. C. O. Fusco-Almeida A. M. Mendes-Giannini M. J. S. (2015). A flow cytometry method for testing the susceptibility of cryptococcus spp. to amphotericin B. Rev. Iberoam. Micol. 32, 159–163. 10.1016/j.riam.2014.06.004 25639695

[B2] Chakrabarti A. Chatterjee S. S. Rao K. L. N. Zameer M. M. Shivaprakash M. R. Singhi S. (2009). Recent experience with fungaemia: change in species distribution and azole resistance. Scand. J. Infect. Dis. 41 (4), 275–284. 10.1080/00365540902777105 19229762

[B3] Chan L. L. Lyettefi E. J. Pirani A. Smith T. Qiu J. Lin B. (2011). Direct concentration and viability measurement of yeast in corn mash using a novel imaging cytometry method. J. Industrial Microbiol. Biotechnol. 38 (8), 1109–1115. 10.1007/s10295-010-0890-7 20960026

[B4] Chaturvedi V. Ramani R. Pfaller M. A. (2004). Collaborative study of the NCCLS and flow cytometry methods for antifungal susceptibility testing of *Candida albicans* . J. Clin. Microbiol. 42 (5), 2249–2251. 10.1128/JCM.42.5.2249-2251.2004 15131203 PMC404628

[B5] Cleveland A. A. Harrison L. H. Farley M. M. Hollick R. Stein B. Chiller T. M. (2015). Declining incidence of candidemia and the shifting epidemiology of candida resistance in two US metropolitan areas, 2008-2013: results from population-based surveillance. PLoS ONE 10 (3), e0120452. 10.1371/journal.pone.0120452 25822249 PMC4378850

[B6] Clinical and Laboratory Standards Institute (CLSI) (2008). Reference method for broth dilution antifungal susceptibility testing of yeasts. approved standard. 3th ed. Wayne, PA: Clinical and Laboratory Standards Institute. Available at: https://clsi.org/media/1461/m27a3_sample.pdf.

[B7] Esfandiary M. A. Farasat A. Rostamian M. Fattahy A. (2012). Study of morphological characteristics, pathogenicity and drug resistance of *Candida glabrata* as increasing opportunistic yeast. Eur. J. Exp. Biol. 2 (4), 948–952.

[B8] Gökahmetoǧlu S. Nedret Koc A. Patiroǧlu T. (2003). Antifungal susceptibility testing of *Candida albicans* by flow cytometry. Empfindlichkeitsprufung an *Candida albicans* mittels durchflusszytometrie. Mycoses 46 (8), 289–293. 10.1046/j.1439-0507.2003.00903.x 12950899

[B9] Horn J. N. Cravens A. Grossfield A. (2013). Interactions between fengycin and model bilayers quantified by coarse-grained molecular dynamics. Biophysical J. 105 (7), 1612–1623. 10.1016/j.bpj.2013.08.034 PMC3822635 24094402

[B10] Kwolek-Mirek M. Zadrag-Tecza R. (2014). Comparison of methods used for assessing the viability and vitality of yeast cells. FEMS Yeast Res. 14 (7), 1068–1079. 10.1111/1567-1364.12202 25154541

[B11] Lockhart S. R. Iqbal N. Cleveland A. A. Farley M. M. Harrison L. H. Bolden C. B. (2012). Species identification and antifungal susceptibility testing of candida bloodstream isolates from population-based surveillance studies in two U.S. cities from 2008 to 2011. J. Clin. Microbiol. 50 (11), 3435–3442. 10.1128/JCM.01283-12 22875889 PMC3486211

[B12] Malina A. Shai Y. (2005). Conjugation of fatty acids with different lengths modulates the antibacterial and antifungal activity of a cationic biologically inactive peptide. Biochem. J. 390 (3), 695–702. 10.1042/BJ20050520 15907192 PMC1199663

[B13] Millard P. J. Roth B. L. Thi H. P. T. Yue S. T. Haugland R. P. (1997). Development of the FUN-1 family of fluorescent probes for vacuole labeling and viability testing of yeasts. Appl. Environ. Microbiol. 63 (7), 2897–2905. 10.1128/aem.63.7.2897-2905.1997 9212436 PMC168585

[B14] Mota S. Alves R. Carneiro C. Silva S. Brown A. J. Istel F. (2015). *Candida glabrata* susceptibility to antifungals and phagocytosis is modulated by acetate. Front. Microbiol. 6, 919–1012. 10.3389/fmicb.2015.00919 26388859 PMC4560035

[B15] Nagayoshi Y. Miyazaki T. Shimamura S. Nakayama H. Minematsu A. Yamauchi S. (2017). Unexpected effects of azole transporter inhibitors on antifungal susceptibility in *Candida glabrata* and other pathogenic Candida species. PLoS ONE 12 (7), e0180990. 10.1371/journal.pone.0180990 28700656 PMC5507446

[B16] Naicker S. D. Shuping L. Zulu T. G. Mpembe R. S. Mhlanga M. Tsotetsi E. M. (2023). Epidemiology and susceptibility of *Nakaseomyces* (formerly *Candida*) *glabrata* bloodstream isolates from hospitalised adults in South Africa. Med. Mycol. 61 (6), myad057. 10.1093/mmy/myad057 37336590

[B17] Nash E. E. Peters B. M. Lilly E. A. Noverr M. C. Fidel P. L. (2016). A murine model of *Candida glabrata* vaginitis shows no evidence of an inflammatory immunopathogenic response. PLoS ONE 11 (1), e0147969. 10.1371/journal.pone.0147969 26807975 PMC4726552

[B18] Pappas P. G. Lionakis M. S. Arendrup M. C. Ostrosky-Zeichner L. Kullberg B. J. (2018). Invasive candidiasis. Nat. Rev. Dis. Prim. 4, 18026–18120. 10.1038/nrdp.2018.26 29749387

[B19] Pina-Vaz C. Sansonetty F. Rodrigues A. G. Costa-Oliveira S. Tavares C. Martinez-De-Oliveira J. (2001). Cytometric approach for a rapid evaluation of susceptibility of *Candida* strains to antifungals. Clin. Microbiol. Infect. 7 (11), 609–618. 10.1046/j.1198-743X.2001.00307.x 11737085

[B20] Pore R. S. (1994). Antibiotic susceptibility testing by flow cytometry. J. Antimicrob. Chemother. 34 (5), 613–627. 10.1093/jac/34.5.613 7706157

[B21] Ramachandran R. Ramesh S. Ramkumar S. Chakrabarti A. Roy U. (2018a). Calcium alginate bead-mediated enhancement of the selective recovery of a lead novel antifungal bacillomycin variant. Appl. Biochem. Biotechnol. 186 (4), 917–936. 10.1007/s12010-018-2778-3 29797296

[B22] Ramachandran R. Shrivastava M. Narayanan N. N. Thakur R. L. Chakrabarti A. Roy U. (2018b). Evaluation of antifungal efficacy of three new cyclic lipopeptides of the class bacillomycin from *Bacillus subtilis* RLID 12.1. Antimicrob. Agents Chemother. 62 (1), e01457–17. 10.1128/AAC.01457-17 29038271 PMC5740331

[B23] Ramchandran R. Ramesh S. Aviksha A. Thakur R. Chakrabarti A. R. U. Roy U. (2020). Improved production of two anti-*Candida* lipopeptide homologues co-produced by the wild-type *Bacillus subtilis* RLID 12.1 under optimized conditions. Curr. Pharm. Biotechnol. 21 (5), 438–450. 10.2174/1389201020666191205115008 31804165

[B24] Ramesh S. Madduri M. Rudramurthy S. M. Roy U. (2023). Functional characterization of a bacillus-derived novel broad-spectrum antifungal lipopeptide variant against *Candida tropicalis* and *Candida auris* and unravelling its mode of action. Microbiol. Spectr. 11 (2), e0158322. 10.1128/spectrum.01583-22 36744953 PMC10100908

[B25] Richardson M. Lass-Flörl C. (2008). Changing epidemiology of systemic fungal infections. Clin. Microbiol. Infect. 14 (4), 5–24. 10.1111/j.1469-0691.2008.01978.x 18430126

[B26] Rodrigues C. F. Silva S. Henriques M. (2014). *Candida glabrata*: a review of its features and resistance. Eur. J. Clin. Microbiol. Infect. Dis. 33 (5), 673–688. 10.1007/s10096-013-2009-3 24249283

[B27] Salazar S. B. Wang C. Münsterkötter M. Okamoto M. Takahashi-Nakaguchi A. Chibana H. (2018). Comparative genomic and transcriptomic analyses unveil novel features of azole resistance and adaptation to the human host in *Candida glabrata* . FEMS Yeast Res. 18 (1), 1–11. 10.1093/femsyr/fox079 29087506

[B28] Seyedjavadi S. S. Khani S. Eslamifar A. Ajdary S. Goudarzi M. Halabian R. (2019). The antifungal peptide MCh-AMP1 derived from matricaria chamomilla inhibits *Candida albicans* growth via inducing ROS generation and altering fungal cell membrane permeability. Front. Microbiol. 10, 3150–3210. 10.3389/fmicb.2019.03150 32038583 PMC6985553

[B29] Yan Y. Tan F. Miao H. Wang H. Cao Y. Y. (2019). Effect of shikonin against *Candida albicans* biofilms. Front. Microbiol. 10, 1085–1111. 10.3389/fmicb.2019.01085 31156594 PMC6527961

[B30] Zhang N. Fan Y. Li C. Wang Q. Leksawasdi N. Li F. (2018). Cell permeability and nuclear DNA staining by propidium iodide in basidiomycetous yeasts. Appl. Microbiol. Biotechnol. 102 (9), 4183–4191. 10.1007/s00253-018-8906-8 29572560

