# Supplementary material for: Two promising natural lipopeptides from Bacillus subtilis effectively induced membrane permeabilization in Candida glabrata
Source: Acta Biochim Pol. 2024 Jan 31;71:11999. doi: 10.3389/abp.2024.11999 (PMC11077351; doi:10.3389/abp.2024.11999)
Supplement: Supplementary file 1 [file DataSheet1.pdf]

Supplementary Fig. 1

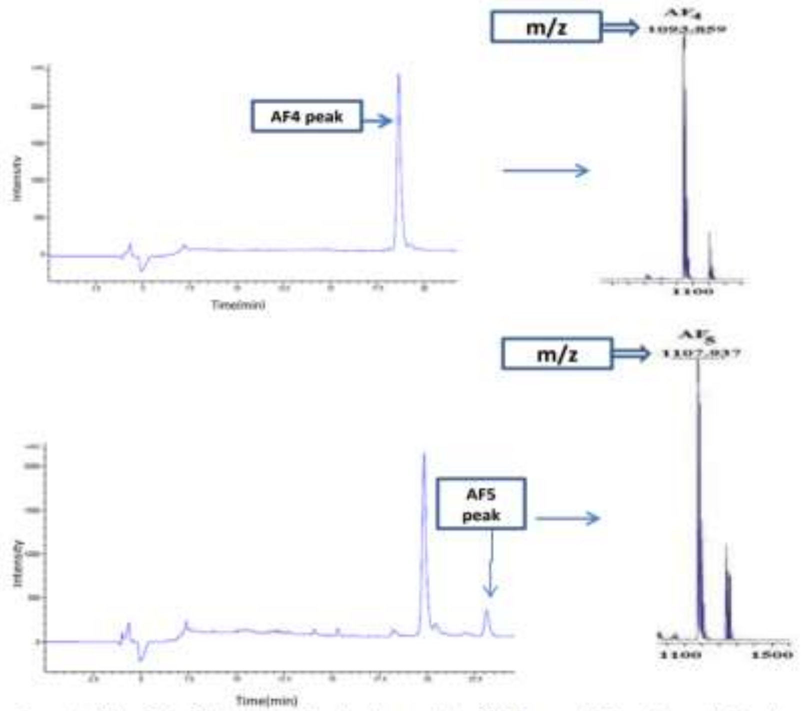

Suppl. Fig. 1. Left panel: A single peak of AF_4 and AF_5 at analytical scale reversed-phase HPLC. Right panel: MALDI-TOF- MS profile showing m/z ratios of HPLC purified lipopeptides AF_4 and AF_5

Supplementary Fig. 2

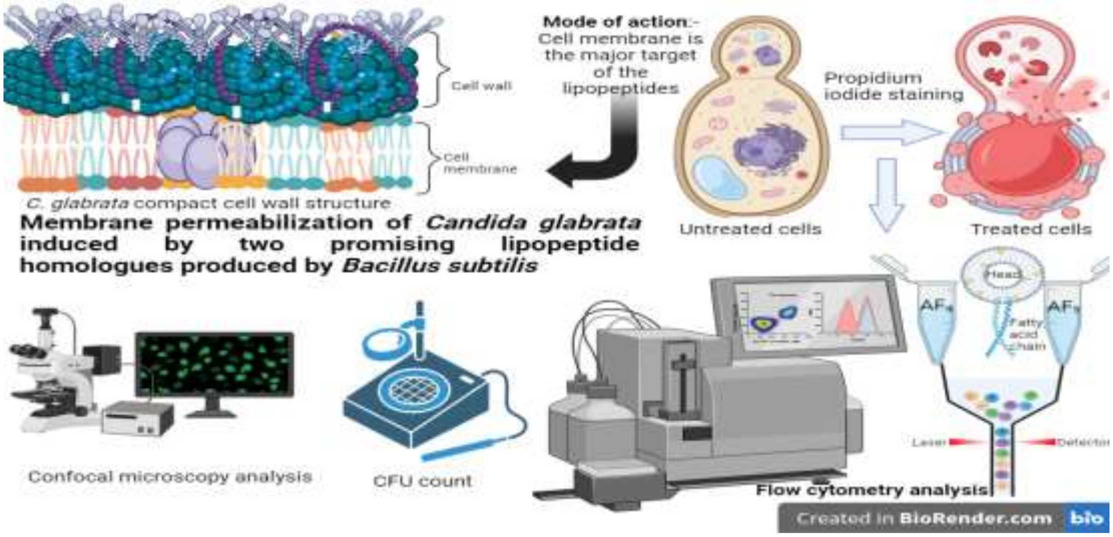

Suppl. Figure 2. A schematic presentation of the preliminary mode of action of the membrane permeabilizing lipopeptides.
